# LPS regulates the expression of glucocorticoid receptor α and β isoforms and induces a selective glucocorticoid resistance in vitro

**DOI:** 10.1186/s12950-017-0169-0

**Published:** 2017-10-16

**Authors:** Maria Luisa Molina, Julia Guerrero, John A. Cidlowski, Héctor Gatica, Annelise Goecke

**Affiliations:** 1 Rheumatology Section, Internal Medicine Department, Clinical Hospital, University of Chile, Santiago, Chile; 2 Physiology and Biophysics Disciplinary Program, ICBM, Faculty of Medicine, University of Chile, Santiago, Chile; 3 Department of Health and Human Services, National Institute of Environmental Health Sciences, National Institutes of Health, Research Triangle Park, NC USA

**Keywords:** Lipopolysaccharide, Glucocorticoid receptor, Glucocorticoid resistance, Acute inflammation

## Abstract

**Background:**

This study was aimed to evaluate the effect of LPS in glucocorticoid receptor (GR) isoforms expression on different cell lines and PBMC from healthy donors in vitro and glucocorticoid sensitivity of PBMC in vitro.

**Methods:**

U-2 OS cell lines expressing GR isoforms, different cell lines (CEM, RAJI, K562 and HeLa) or PBMC from healthy donors, were cultured or not with LPS. The expression of GRα and GRβ was evaluated by Western blot. Glucocorticoid sensitivity was evaluated in PBMC treated with LPS, testing genes which are transactivated or transrepressed by glucocorticoid. For transactivated genes (MKP1, FKBP5) PBMC were treated with Dexamethasone 100 nM for 6 h. The mRNA expression was measured by RT-PCR. For transrepressed genes (IL-8, GM-CSF), PBMC were cultured in Dexamethasone 100 nM and LPS 10 μg/ml for 6 h and protein expression was measure by ELISA.

**Results:**

GR isoforms were induced in U-2 OS cells with a greater effect on GRα expression. Both isoforms were also induced in CEM cells with a tendency to a greater effect on GRβ. LPS induced only the expression of GRα in Raji and HeLa cells, and in PBMC, with no effect in K562 cells. LPS induced a loss of glucocorticoid inhibitory effect only on the secretion of GM-CSF.

**Conclusion:**

LPS in vitro differentially modulates the expression of GR isoforms in a cell specific manner. In PBMC from healthy donors LPS induces an approximately two times increase in the expression of GRα and a loss of the glucocorticoid inhibitory effect on the secretion of GM-CSF, without affecting other glucocorticoid responses evaluated.

**Electronic supplementary material:**

The online version of this article (10.1186/s12950-017-0169-0) contains supplementary material, which is available to authorized users.

## Background

Glucocorticoids (GC) have been used as anti-inflammatory and immunosuppressive agents [1]. However, the sensitivity to GC actions varies among individuals and even in the same patient at different time points. The mechanisms mediating changes in GC sensitivity are not completely understood [2]. A better understanding of the factors that could influence the cell response to GC is relevant to adapt GC therapy to different requirements in patients suffering autoimmune/inflammatory diseases.

The GC actions are mediated through the activation of intracellular glucocorticoid receptors (GR)***.*** The genomic structure of the single GR gene described in humans consists of 9 exons. Alternative splicing of exon 9 (exon 9α or 9β) results in the synthesis of 2 homologous mRNAs and protein isoforms, termed GRα and GRβ [3]. After GC binds GRα, it binds as a homodimer to DNA, regulating the expression of the linked gene. GRα can also modulate gene expression by interacting with other transcription factors such as AP-1 and NF-κB, exerting most of the anti-inflammatory actions by this mechanism [4–7]. GRβ differs from GRα only in its carboxi-terminus sequence. This difference renders to GRβ a role as a dominant negative inhibitor of GRα activity. Therefore, a relative over-expression of the GRβ isoform could play a role in the regulation of target cell sensitivity to GC [8, 9]. Indeed, studies have demonstrated that GRβ expression is elevated in patients with chronic inflammatory diseases associated with GC resistance e.g. asthma, inflammatory bowel disease and nasal polyps [10–14].

It has long been proposed that infectious episodes could trigger relapses in autoimmune diseases [15, 16]. Our group has demonstrated a transient increased expression of GRβ in peripheral blood mononuclear cells (PBMC) from septic patients. We also showed that septic serum influence GC sensitivity, favoring the expression of GRβ in association with a GC resistant state [17, 18]. Number of factors, present in serum from septic patients, such as cytokines, bacterial products and GC, either by themselves or in combination could be responsible for these effects.

Webster et al. [19] showed that TNF-α and IL-1 induced the expression of both GR isoforms mRNA in HeLa and CEM-C7 cell lines, but the increase of the expression of GRβ was greater than GRα in those conditions. Other proinflammatory cytokines has also been reported to increase the expression of GRβ in vitro, such as IL-7, IL-18, and the combination of IL-2 and IL-4 in PBMC, and IL-8 in human neutrophils [13, 20, 21]. Interestingly, these cytokine treatments resulted in decreased sensitivity to GC in these cell types [13, 19–21]. Also, bacterial superantigens and bacterial toxins have been reported to increase the expression of GRβ [22, 23].

Septic serum also contains fragments of bacteria which are recognized by innate immune system [24]. In our study, serum samples were obtained mostly from patients where a gram-negative infection was either suspected, due to the primary septic source or demonstrated by culture [18]. TLR4 recognizes lipopolysaccharides (LPS) present in gram-negative bacteria leading to the subsequent induction of the initial inflammatory response [25]. Therefore, the influence of septic serum on GR and GC cell sensitivity in our study could have been the consequence of the activation of TLR4 by LPS.

It is known that GC secreted endogenously by the adrenal gland restrict the responses to TLR [26]. Multiple mechanisms have been described for the GC interference with TLRs signaling [27, 28]. However, if TLR activation influences GR expression or GC sensitivity, has been scarcely studied. Indirect evidence of an effect of TLR activation on GR isoforms expression comes from two studies were an up regulation of GRβ expression was reported in vivo after the exposure to TLR4 agonist. One, in a septic animal model induced by LPS injection [29], and the other studying children with respiratory syncytial virus (RSV) infection, another known agonist of TLR4 [30]. However, it should be noted that after the induction of sepsis, and in children with RSV infection, changes in metabolic parameters, as well as cytokines, toxins and stress hormones levels are expected. Therefore, it is unclear the cause of GR isoforms modulation in both situations. The effect of LPS has been tested in vitro in bone marrow-derived macrophages and nasal mucosa fibroblasts with contradictory results, but it has not been evaluated on PBMC or lymphocytes in vitro [31, 32].

In this report, we evaluated the effect of LPS, a TLR4 agonist, on GRα and GRβ expression in different cell lines and PBMC from healthy donors in culture. We also explore the effect of LPS on GC cell sensitivity in vitro.

## Methods

### Cell culture and transfection

Human cell lines (CEM, K562, Raji and HeLa) were obtained from American Type Culture Collection, U-2 OS from Dr. Marcel Shaaf, and PBMC from healthy donors. CEM, K562, Raji cells and PBMC were grown in RPMI-1640 medium (GIBCO), supplemented with 10% fetal calf serum (FCS), 4 mM L-glutamine,100 U/ml penicillin, 100 μg/ml streptomycin at 37 °C in a 5% CO_2_ humidified atmosphere. HeLa and U-2 OS cells were grown in DMEM-F12 medium (Invitrogen Life Technologies) containing 10% FCS, glutamine, penicillin and streptomycin as described above.

Cell viability was >95%, determined by trypan blue exclusion staining, in cultures in the presence or not of LPS.

U-2 OS cell lines stably expressing human GRα or GRβ isoform were generated as previously described [33].

### Mononuclear cell extraction

Peripheral venous blood samples (30 ml) from healthy volunteers were collected and PBMC were isolated by density gradient centrifugation (Ficoll-Paque Plus, Amersham Bioscience). Written informed consent was previously obtained from all volunteers, according to the Declaration of Helsinki.

### Stimulation with LPS

U-2 OS, CEM, K562, Raji, HeLa cells and PBMC (2 × 10^6^ cells/ml) were cultured with LPS from *E.coli* serotype O111:B4 (Sigma-Aldrich) at 0.1, 1, 10 μg/ml; or only the vehicle as control, for 24 h.

### GC sensitivity assay

PBMC treated previously with or without LPS for 24 h were washed twice with PBS 1X and then GC sensitivity was evaluated by measuring: 1) Dexamethasone (DEX) induction of FKPB5 and MKP1 mRNA expression. Briefly, PBMC were cultured in the presence or absence of DEX 100 nM for 6 h, then FKPB5 and MKP1 mRNA expression was evaluated by qPCR. 2) DEX inhibition of LPS induced IL-8 and GM-CSF release by ELISA. Briefly PBMC were re-stimulated with 10 μg/ml of LPS for 6 h in presence or absence of DEX 100 nM. After incubation, cells were centrifuged and supernatant were collected and stored until the ELISA assay.

### Western blot analysis

Cells were harvested and lysed using RIPA buffer (50 mM Tris pH 8.0, 150 mM NaCl, 1% Tween 20, 0.5% SDS) containing protease inhibitors (0.1 mM phenylmetthylsulfonyl fluoride, 1 μg/ml aprotinin, 1 μM pepstatin, 1 μM leupeptin). Proteins (50μg) were resolved by electrophoresis through 10% polyacrylamide gels and transferred to nitrocellulose membranes as previously described [18]. Membranes were blocked in PBS 1X containing 10% nonfat dry milk and 0,1% Tween 20, and incubated overnight at 4°C with a purified polyclonal rabbit anti-hGR antiserum (dilution 1:1000 to 1:2000) [34], specific polyclonal rabbit anti-GRα antibodies (dilution 1:200) (GR P-20: sc1002; Santa Cruz Biotecnology Inc., Santa Cruz, CA, USA), or specific polyclonal rabbit anti-GRβ antibodies (dilution 1:500) (PA3–514; Affinity BioReagents, Golden, CO, USA.); and 1 h at room temperature with mouse monoclonal anti β-actin (dilution 1:40,000) (Santa Cruz Biotecnology, Inc). Secondary biotinilated polyclonal antibodies against rabbit or mouse IgG (DAKO) were used for GR and β-actin detection, respectively. Films were densitometrically analyzed using Image J (Scion Corp.) and normalized to β-actin as loading control.

### Quantitative real-time PCR analysis

Total RNA was isolated using Trizol^®^ Reagent (Invitrogen) according to the manufacturer’s instructions. RNA concentration was determined by spectrophotometry and integrity of RNA by agarose gel electrophoresis. One μg of RNA was reverse transcribed using ImProm-II™ Reverse Transcription System (Promega). PCR analysis was performed in triplicate for each experiment with Brilliant SYBR Green in the Mx3000P® real-time PCR system (Stratagene, La Jolla, CA). The primers used for RT-PCR were MKP1: 5′-GGTTCTTCTAGGAGTAGACA-3′ (upstream) and 5′-GTACATCAAGTCCATCTGAC-3′ (downstream); FKPB5: 5′-TTGAGGAGGGGCCGAGTT-3′ (upstream) and 5′-AAAAGGCCAAGGAGCACAAC-3′ (downstream); h18S (housekeeping): 5′-GATATGCTCATGTGGTGTTG-3′ (upstream) and 5′-AATCTTCTTCAGTCGCTCCA-3′ (downstream). Relative mRNA levels for each sample were quantified using the threshold cycle (C_t_) approach and normalized with respect to h18S RNA.

### IL-8 and GM-CSF release quantification

Immunoreactive IL-8 and GM-CSF were determined on the supernatants of PBMC from the GC sensitivity assay by ELISA according to the manufacturer’s instructions (Pierce Biotechnology, Inc.). Data are presented as the average of triplicate results. The lower limit of detection was 2 pg/ml for both assays.

### Statistic analysis

All data are presented as medians with interquartile range and range of at least three independent experiments. Data were analyzed using Student’s t test for normally distributed data and the Mann-Whitney test for nonparametric data, considering a *p*-*value* < 0.05 statistically significant.

## Results

### LPS regulates the expression of GRα and GRβ isoforms in U-2 OS transfected cells

**Fig. 1:**
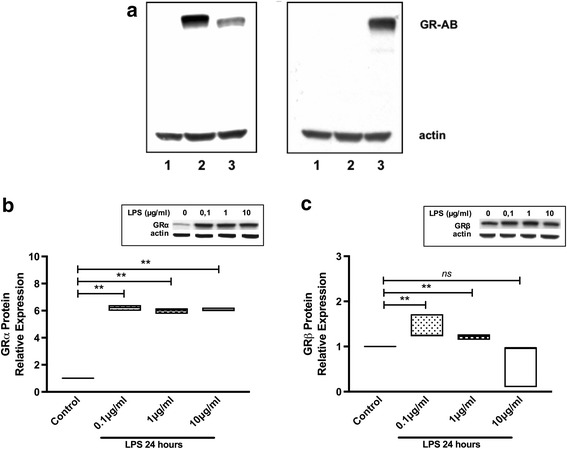
LPS regulates the expression of GRα and GRβ isoforms in U-2 OS transfected cells. **a**. Western blot of total proteins of parental U-OFF cells (line 1), U-2 OS alpha cells (line 2) and U-2 OS beta cells (line 3) are shown. On the left panel, a common antibody to GR was used and on the right panel a specific GRβ antibody was used. U2-OS alpha (**b**) and US-OS beta (**c**) cells were stimulated with LPS for 24 h. The expression of GRα and GRβ was determined by Western blot. The median values of three different experiments, plotted as values relative to control are shown. * *p* < 0.05 and ** *p* < 0.01

Western blot analysis of total proteins from human osteosarcoma cell line lacking endogenous GR (parental U-OFF cells, line 1), transfected with GRα (U-2 OS alpha, line 2) or GRβ (U-2 OS beta, line 3) using different antiGR antibodies is showed in Fig. 1a. When an antibody common to both GR isoforms was used, a protein of ~ 90–95 kDa was detected in cells stably expressing GRα or GRβ isoform, but not in parental U-OFF cells. When an antibody specific for GRβ was used, a protein of ~ 90 kDa was identified only in U-2 OS beta cells.

To evaluate whether LPS stimulation modified GRα or GRβ protein expression in these cells, U-2 OS alpha or U-2 OS beta cells were cultured with different concentrations of LPS (0.1, 1 or 10 μg/ml). After 24 h the expression of GRα and GRβ was evaluated by Western blot with specific antibodies for each isoform. Figure 1b shows that all concentrations of LPS induced at least a significant 6 fold increased expression of GRα (median 6.23, range 5.99–6.41 for LPS 0.1 μg/ml, median 6.04, range 5.76–6.18 for LPS 1 μg/ml, median 6.04, range 5.96–6.23 for LPS 10 μg/ml; *p* = 0.002). LPS at low concentrations, but not LPS 10 μg/ml, induced a significant increase of smaller magnitude in GRβ protein expression (LPS 0.1μg/ml: median 1.25, range 1.23–1.72; LPS 1μg/ml: median 1.23, range 1.15–1.27; *p* = 0.002) (Fig. 1c).

### LPS regulates the expression of GRα and GRβ isoforms in immune and epithelial cell lines

**Fig. 2:**
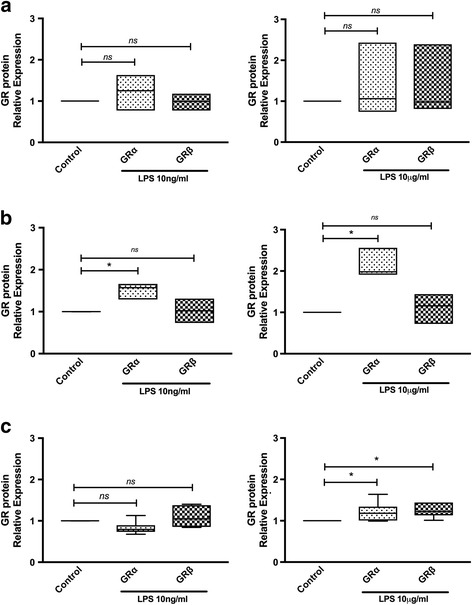
LPS regulates the expression of GRα and GRβ isoforms in immune cell lines. K562 (**a**), Raji (**b**), CEM (**c**) cells were cultured with LPS for 24 h. The expression of GRα and GRβ was determined by Western blot. The median values of three different experiments, plotted as values relative to control are shown. * *p* < 0.05 and ** *p* < 0.01

To assess whether LPS modulates the expression of GR isoforms in cells constitutively expressing this receptor, a monocytic cell line (K562) and two human cell lines of lymphoid origin (CEM and Raji) were used. These cells were treated or not with 10 ng/ml or 10 μg/ml of LPS for 24 h. As shown in Fig. 2, LPS treatment induced a different response in the human immune cells studied. In K562 there was no change in the expression of GR isoforms at 10 ng/ml and 10 μg/ml of LPS (Fig. 2a). In Raji cells there was a significant increase in only GRα expression at both concentrations used (LPS 10 ng/ml: median 1.57, range 1.29–1.66, *p* = 0.01; LPS 10 μg/ml: median 1.98, range 1.91–2.55, *p* = 0.03) (Fig. 2b). In CEM cells there was an increase in the expression of both GR isoforms, being only statistically significant at LPS 10 μg/ml (GRα: median 1.18, range 0.99–1.63, *p* = 0.002; GRβ: median 1.38, range 1.10–1.55, *p* = 0.002) (Fig. 2c).

In HeLa cells, an epithelial cell line, there was a significant increase only in GRα expression (median 1.86, range 1.02–2.37, *p* = 0.002), with no change in GRβ expression when treated with 10 μg/ml of LPS for 24 h (Additional file 1).

### LPS regulates the expression of GRα in PBMC from healthy individuals

**Fig. 3:**
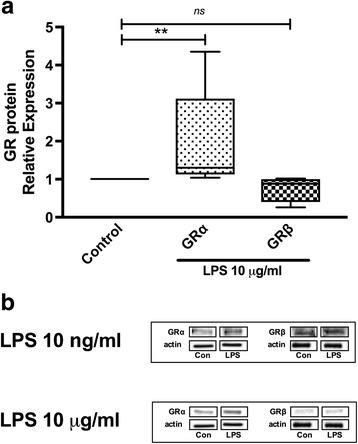
LPS regulates the expression of GRα in PBMC from healthy individuals. PBMC from healthy individuals were cultured with LPS for 24 h. The expression of GRα and GRβ was determined by Western blot. **a** The median values of three different experiments, plotted as values relative to control are shown. * *p* < 0.05 and ** *p* < 0.01. **b** Western blot of total proteins of PBMC stimulated at different concentrations of LPS are shown

We examined whether LPS modulates de expression of GR proteins isoforms in PBMC from healthy individuals. As shown in Fig. 3a, LPS 10 μg/ml treatment significantly increased GRα protein expression (median 1.95, range 1.04–4.35, *p* = 0.007), while GRβ protein expression was not modified. When PBMC were stimulated with LPS 10 ng/ml, a similar result was obtained as mentioned before (Fig. 3b).

### LPS modifies DEX sensitivity depending on the gene studied

**Fig. 4:**
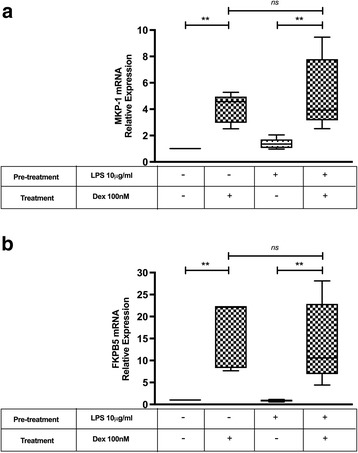
GC Sensitivity Assay in Transactivated Genes. PBMC from healthy individuals were cultured for 24 h with or without LPS, follow for Dex 100 nM for 6 h. MKP1 (**a**) and FKPB5 (**b**) mRNA expression was analyzed by RT-PCR. The median values of three different experiments, plotted as values relative to control, are shown. * *p* < 0.05 and ** *p* < 0.01

To assess whether LPS modify DEX sensitivity in PBMC from healthy individuals, we evaluated genes known to be transactivated o transrepressed by GC. The expression of two genes known to be up regulated by DEX, MKP-1 and FKPB5 were first tested. PBMC were stimulated or not with LPS 10 μg/ml for 24 h, followed by 6 h of DEX 100 nM. DEX induced the expression of MKP-1 and FKPB5 mRNA in PBMC previously treated or not with LPS, but LPS pretreatment did not modify the inducing effect of DEX on MKP-1 (Control 4.82±1.1 vs LPS 3.76±1.6 fold increase, *p* = 0.66) (Fig. 4a) and FKPB5 (Control 16.69±7.6 vs LPS 15.62±8.5 fold increase, *p* = 0.95) mRNA expression (Fig. 4b).

**Fig. 5:**
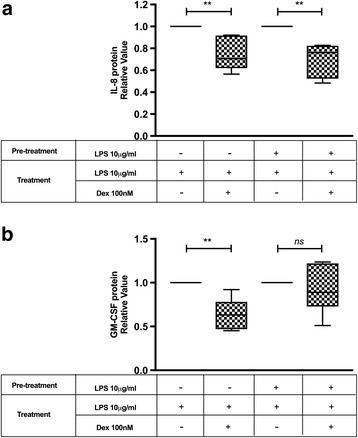
GC Sensitivity Assay in transrepressed genes. PBMC from healthy individuals were cultured for 24 h with or without LPS, follow by DEX 100 nM and LPS 10 μg/ml for 6 h. IL-8 (**a**) and GM-CSF (**b**) content in the culture media was measure by ELISA. The median values of three different experiments, plotted as values relative to control are shown. * *p* < 0.05 and ** *p* < 0.01

To evaluate LPS effect in DEX sensitivity in genes known to be down-regulated by GC, we test DEX capacity to inhibit LPS induced IL-8 and GM-CSF protein expression. PBMC from healthy individuals were first stimulated or not with LPS 10 μg/ml for 24 h and then re-stimulated for 6 h with LPS 10 μg/ml and DEX 100 nM. As show in Fig. 5, DEX inhibited LPS induced expression of GM-CSF when PBMC were not pretreated with LPS (0.64 ±0.18 fold increase respect to control, *p* = 0.0022), while DEX in PBMC pretreated with LPS did not inhibited LPS induced expression of this cytokine (0.92±0.27 fold increase respect to control, *p* = 0.36) (Fig. 5b). The inhibitory effect did not change in PBMC pretreated with LPS when IL-8 was evaluated (Fig. 5a).

## Discussion

Infectious episodes in patients with autoimmune diseases have been always a challenge. Not only because these patients are prone to more severe infections since they commonly are receiving immunosuppressive drugs such as GC, but also because frequently a flare is triggered. Since GC are important in the treatment of autoimmune diseases and also are a key factor in the response to infections, it is important to understand how an infection can influence the cell response to GCs.

This study has shown that LPS, present in Gam-negative bacteria cell wall, is able to differentially regulate the expression of GRα and GRβ in different cell lines and PBMC from healthy donors in culture. LPS induced both, GRα and GRβ in transfected cells (Fig. 1b and c), but with an approximately four times greater effect on GRα expression. Some other groups have evaluated the effect of LPS on the expression of GR. Haim et al. showed that LPS on bone marrow-derived macrophages induced preferentially the expression of GRβ, resulting in a partial GC resistance [31]. However, Fernández-Bertolín et al., have shown that LPS 10 μg/ml did not modify the expression of GR on nasal mucosa fibroblasts, but it did induce a GC resistance for some but not all GC actions tested [32]. We therefore evaluated the effect of LPS on different cell lines relevant in the immune system. As is shown in Fig. 2, LPS in CEM cells (T cell line) induced both, GRα and GRβ isoforms expression at the same concentrations used by Fernandez-Bertolin et al. [32], with a tendency to a greater effect on GRβ, similar to the results of Haim et al. in macrophages. However LPS induced only the expression of GRα in Raji (B cell line) and HeLa (epithelial cell line) cells (Additional file 1), with no effect in the expression of either isoforms in K562 cells. Therefore, our results and what has been reported previously demonstrate that LPS can affect the expression of the GR isoforms, but this is a cell type specific response.

Since in normal circumstances T cells, B cells and monocytes will coexist when exposed to LPS, influencing each other, we decided to evaluate the effect of LPS on PBMC in vitro. Figure 3 shows that in these conditions there was an almost two time increase in GRα expression with no change in GRβ. This effect is opposite to the effect of serum from septic patients, which induced preferentially GRβ expression on PBMC from healthy donors [19]. There are several possible explanations for this apparent contradiction. For instance, LPS is just one of the components of the serum of patients with septic states. In an inflammatory process induced by LPS there are many mediators such as cytokines, chemokines, lipid mediators, and reactive oxygen species among others that participate in the inflammatory process [35]. Any of these components could modulate the expression of GR isoforms, and even depending on the concentration balance of these mediators, different responses could be elicited.

The GRα commonly has been described as the active GR isoform which mediates most of known GC actions, while GRβ can exert a negative dominant action over GRα. Therefore, we expected that LPS would induced an increase sensitivity to GC actions on PBMC. Surprisingly our results showed that LPS treatment induced a loss of GC inhibitory effect on the production of GM-CSF. This resistance to GC effects was not generalized, since the inhibitory effect on the production of IL-8 or the induction of MKP-1 and FKPB-5 were maintained (Figs. 4 and 5). The effects of GC even when mediated by GR, are not only dependent on the ratio of expression of GRα and β. They can also be altered by nuclear translocation, changes in GR affinity, phosphorylation, among others [6, 36]. Therefore, it is possible that one or several of this factors could be involved in the GC loss of inhibition of GM-CSF induced by LPS. Indeed, in NM fibroblast, Fernández-Bertolin et al. showed that LPS 10 μg/ml treatment lead to a reduced dexamethasone-induced GRα nuclear translocation and a decreased GILZ expression without changing the GRα/GRβ expression and maintaining dexamethasone’s induction effect on MKP-1 and GILZ expression. Therefore, they also showed a GC resistant state limited to some but not all GC effects which was not associated to a change on GR expression [32].

It is also interesting that Armstrong et al., showed that even when most cytokines maintain a normal sensitivity to GC actions in macrophages from COPD patients a GC resistance was demonstrated for GM-CSF [37].

## Conclusions

In summary our study showed that LPS in vitro modulates the expression of GRα and GRβ isoforms in a cell specific manner. In PBMC from healthy donor LPS induces the expression of GRα with no change of expression of GRβ. It also induces a loss of GC inhibitory effect on the secretion of GM-CSF without affecting GC induction of MKP1 or FKBP5.

From our results as well as others, it is important to note that GCs effects can be modulated in a cell and effect specific manner. Therefore, it is not possible when a particular change of GC effects is observed under certain condition, to extrapolate those findings to other GC actions.

## Additional file

Additional file 1: LPS regulates the expression of GRα and GRβ isoforms in a epithelial cell line. HeLa cells were cultured with LPS for 24 h. The expression of GRα and GRβ was determined by Western blot. The median values of three different experiments, plotted as values relative to control are shown. * *p* < 0.05 and ** *p* < 0.01. (TIFF 128 kb)

## Abbreviaitons

DEX: Dexamethasone
GC: Glucocorticoids
GR: Glucocorticoid receptor
LPS: Lipopolysaccharides
PBMC: Peripheral blood mononuclear cells
RSV: Respiratory syncytial virus

## References

[1] Buttgereit F, Straub RH, Wehking M, Burmester GR (2004). Glucocorticoids in the treatment of rheumatic diseases: an update on the mechanisms of action. Arthritis Rheum.

[2] Gross KL, Lu NZ, Cidlowski JA (2009). Molecular mechanisms regulating glucocorticoid sensitivity and resistance. Mol Cell Endocrinol.

[3] Hollenberg SM, Weinberger C, Ong ES, Cerelli G, Oro A, Lebo R (1985). Primary structure and expression of a functional human glucocorticoid receptor cDNA. Nature.

[4] Pujols L, Mullol J, Torrego A, Picado C (2004). Glucocorticoid receptors in human airways. Allergy.

[5] De Bosscher K, Vanden Berghe W, Haegeman G (2003). The interplay between the glucocorticoid receptor and nuclear factor-kappaB or activator protein −1: molecular mechanisms of gene repression. Endocr Rev.

[6] Leung DY, Bloom JW (2003). Update on glucocorticoid action and resistance. J Allergy Clin Immunol.

[7] Busillo JM, Cidlowski JA (2013). The five Rs of glucocorticoid action during inflammation: ready, reinforce, repress, resolve, and restore. Trends Endocrinol Metab.

[8] Oakley RH, Jewell CM, Yudt MR, Bofetiado D, Cidlowski JA (1999). The dominant negative activity of the human glucocorticoid receptor beta isoform. Specificity and mechanisms of action. J Biol Chem.

[9] Yudt MR, Jewell CM, Bienstock RJ, Cidlowski JA (2003). Molecular origins for the dominant negative function of human glucocorticoid receptor beta. Mol Cell Biol.

[10] Hamid QA, Wenzel SE, Hauk PJ, Tsicopoulos A, Wallaert B, Lafitte JJ (1999). Increased glucocorticoid receptor beta in airway cells of glucocorticoid-insensitive asthma. Am J Respir Crit Care Med.

[11] Goleva E, Li LB, Eves PT, Strand MJ, Martin RJ, Leung DY (2006). Increased glucocorticoid receptor beta alters steroid response in glucocorticoid-insensitive asthma. Am J Respir Crit Care Med.

[12] Honda M, Orii F, Ayabe T, Imai S, Ashida T, Obara T (2000). Expression of glucocorticoid receptor beta in lymphocytes of patients with glucocorticoid-resistant ulcerative colitis. Gastroenterology.

[13] Orii F, Ashida T, Nomura M, Maemoto A, Fujiki T, Ayabe T (2002). Quantitative analysis for human glucocorticoid receptor alpha/beta mRNA in IBD. Biochem Biophys Res Commun.

[14] Hamilos DL, Leung DY, Muro S, Kahn AM, Hamilos SS, Thawley SE (2001). GRbeta expression in nasal polyp inflammatory cells and its relationship to the anti-inflammatory effects of intranasal fluticasone. J Allergy Clin Immunol.

[15] Samarkos M, Vaiopoulos G (2005). The role of infections in the pathogenesis of autoimmune diseases. Curr Drugs Targets Inflamm Allergy.

[16] Doria A, Sarzi-Puttini P, Shoenfeld Y (2008). Infections, rheumatism and autoimmunity: The conflicting relationship between humans and their environment. Autoimmun Rev.

[17] Goecke A, Guerrero J (2006). Glucocorticoid receptor beta in acute and chronic inflammatory conditions: clinical implications. Immunobiology.

[18] Guerrero J, Gatica HA, Rodriguez M, Estay R, Goecke IA (2013). Septic serum induces glucocorticoid resistance and modifies the expression of glucocorticoid isoforms receptors: a prospective cohort study and in vitro experimental assay. Crit Care.

[19] Webster JC, Oakley RH, Jewell CM, Cidlowski JA (2001). Proinflammatory cytokines regulate human glucocorticoid receptor gene expression and lead to the accumulation of the dominant negative beta isoform: a mechanism for the generation of glucocorticoid resistance. Proc Natl Acad Sci U S A.

[20] Leung DY, Hamid Q, Vottero A, Szefler SJ, Surs W, Minshall E (1997). Association of glucocorticoid insensitivity with increased expression of glucocorticoid receptor beta. J Exp Med.

[21] Strickland I, Kisich K, Hauk PJ, Vottero A, Chrousos GP, Klemm DJ (2001). High constitutive glucocorticoid receptor beta in human neutrophils enables them to reduce their spontaneous rate of cell death in response to corticosteroids. J Exp Med.

[22] Fakhri S, Tulic M, Christodoulopoulos P, Fukakusa M, Frenkiel S, Leung DY (2004). Microbial superantigens induce glucocorticoid receptor beta and steroid resistance in a nasal explant model. Laryngoscope.

[23] Fakhri S, Christodoulopoulos P, Tulic M, Fukakusa M, Frenkiel S, Leung DY (2003). Role of microbial toxins in the induction of glucocorticoid receptor beta expression in an explant model of rhinosinusitis. J Otolaryngol.

[24] Van Amersfoort ES, Van Berkel TJ, Kuiper J (2003). Receptors, mediators, and mechanisms involved in bacterial sepsis and septic shock. Clin Microbiol Rev.

[25] Poltorak A, He X, Smirnova I, Liu MY, Van Huffel C, Du X (1998). Defective LPS signaling in C3H/HeJ and C57BL/10ScCr mice: mutations in Tlr4 gene. Science.

[26] Chrousos GP (1995). The hypothalamic-pituitary-adrenal axis and immune-mediated inflammation. N Engl J Med.

[27] Abraham SM, Lawrence T, Kleiman A, Warden P, Medghalchi M, Tuckermann J (2006). Antiinflammatory effects of dexamethasone are partly dependent on induction of dual specificity phosphatase 1. J Exp Med.

[28] Ogawa S, Lozach J, Benner C, Pascual G, Tangirala RK, Westin S (2005). Molecular determinants of crosstalk between nuclear receptors and toll-like receptors. Cell.

[29] Kamiyama K, Matsuda N, Yamamoto S, Takano K, Takano Y, Yamazaki H (2008). Modulation of glucocorticoid receptor expression, inflammation, and cell apoptosis in septic guinea pig lungs using methylprednisolone. Am J Physiol Lung Cell Mol Physiol.

[30] Diaz PV, Pinto RA, Mamani R, Uasapud PA, Bono MR, Gaggero AA (2012). Increased expression of the glucocorticoid receptor β in infants with RSV bronchiolitis. Pediatrics.

[31] Haim YO, Unger ND, Souroujon MC, Mittelman M, Nuemann D (2014). Resistance of LPS-activated bone marrow derived macrophages to apoptosis mediated by dexamethasone. Sci Rep.

[32] Fernández-Bertolín L, Mullol J, Fuentes-Prado M, Roca-Ferrer J, Alobid I, Picado C (2015). Effect of lipololysaccharide on glucocorticoid receptor function in control nasal mucosa fibroblasts and in fibroblasts from patients with chronic rhinosinusitis with nasal polyps and asthma. PLoS One.

[33] Lu NZ, Cidlowski JA (2005). Translational regulatory mechanisms generate N-terminal glucocorticoid receptor isoforms with unique transcriptional target genes. Mol Cell.

[34] Cidlowski JA, Bellinghan DL, Powell-Oliver FE, Lubahn DB, Sar M (1990). Novel antipeptide antibodies to the human glucocorticoid receptor: recognition of multiple receptor forms in vitro and distinct localization of cytoplasmic and nuclear recpetors. Mol Endocrinol.

[35] Cohen J (2002). The immunopathogenesis of sepsis. Nature.

[36] Kadmiel M, Cidlowski JA (2013). Glucocorticoid receptor signaling in health and disease. Trends Pharmacol Sci.

[37] Armstrong J, Sargent C, Singh D (2009). Glucocorticoid sensitivity of lipopolysaccharide-stimulated chronic obstructive pulmonary disease alveolar macrophages. Clin Exp Immunol.

