# Supplementary figures and images for: LPS regulates the expression of glucocorticoid receptor α and β isoforms and induces a selective glucocorticoid resistance in vitro
Source: J Inflamm (Lond). 2017 Oct 16;14:22. doi: 10.1186/s12950-017-0169-0 (PMC5644185; doi:10.1186/s12950-017-0169-0)

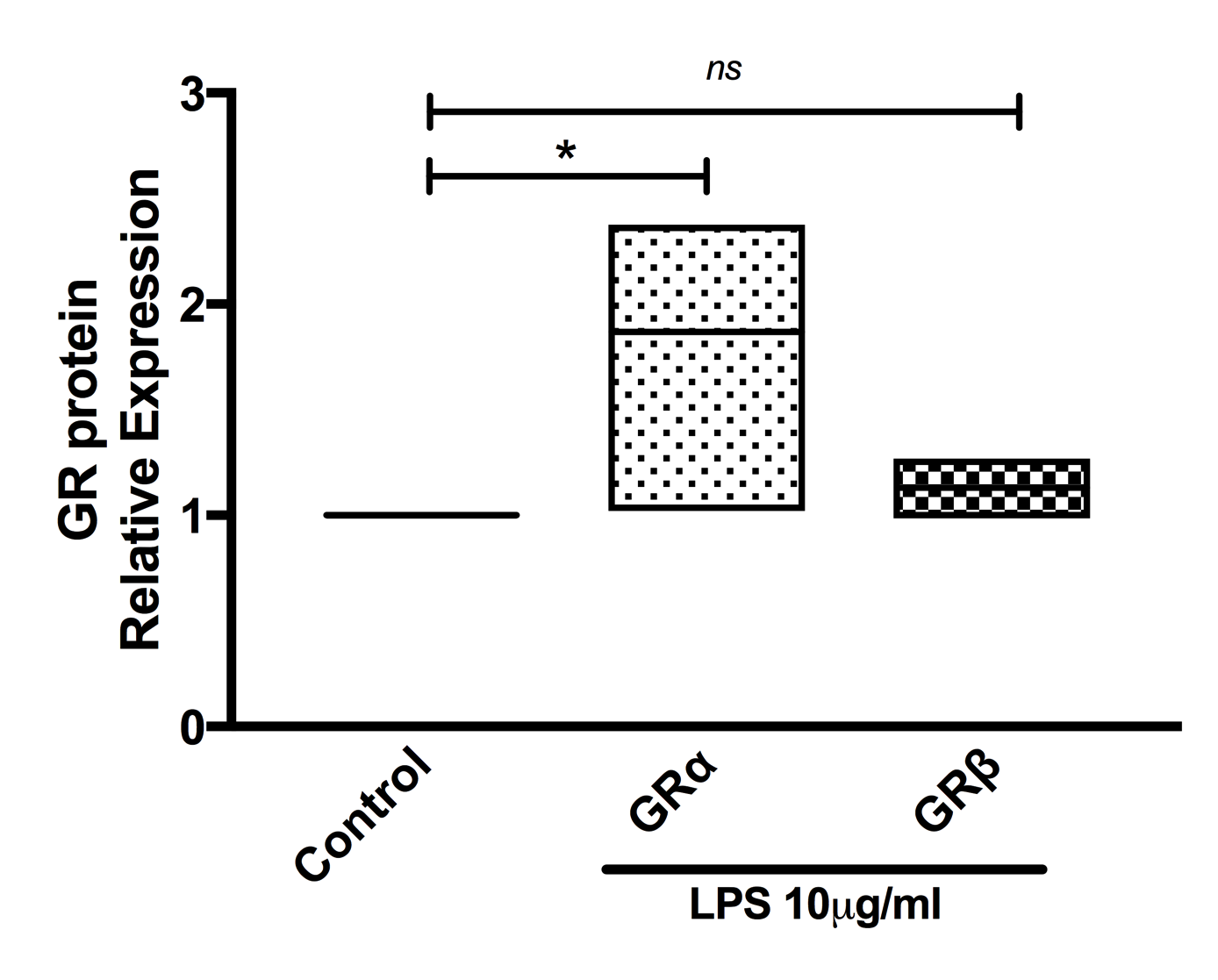

Supplement: Additional file 1: — LPS regulates the expression of GRα and GRβ isoforms in a epithelial cell line. HeLa cells were cultured with LPS for 24 h. The expression of GRα and GRβ was determined by Western blot. The median values of three different experiments, plotted as values relative to control are shown. * p < 0.05 and ** p < 0.01. (TIFF 128 kb) [file 12950_2017_169_MOESM1_ESM.tiff]
